# Multi-month fouling control on UV-emitting glass: performance, energy dependence, and insights into microbial community drivers

**DOI:** 10.1016/j.bioflm.2026.100407

**Published:** 2026-10-27

**Authors:** Leila Alidokht, Karina A. Chavarria, Dylan Eggers, Kelli Hunsucker, Mariana Lanzarini-Lopes

**Affiliations:** a Environmental and Water Resource Engineering, Department of Civil and Environmental Engineering, University of Massachusetts, Amherst, MA, USA; b Center for Corrosion and Biofouling Control, Florida Institute of Technology, Melbourne, FL, USA

**Keywords:** UV-emitting glass, Antifouling, Marine biofouling, Microbial community succession, Biofilm, UV LED

## Abstract

UV-emitting glass (UEG) offers a promising solution to control biofouling on submerged surfaces without the need for chemicals, yet its long-term efficacy under real marine conditions has not been established. In this study, UEG lenses designed for multi-month marine submersion were deployed for three months at Port Canaveral, FL, driven by 265 nm UV-LEDs at a 30 min on/off duty cycle to emit ∼55 μW/cm^2^ when submerged. The results showed that UEG surfaces maintain fouling coverage below 15% throughout the entire 12-week deployment, while controls reached 100% surface coverage within two weeks, a disparity that persisted and widened over the full deployment period. Control biofilm volume and thickness increased nearly four-fold between months one and three, producing dense barnacle- and bryozoan-dominated assemblages, whereas UEG surfaces remained near baseline. Microbially, controls followed canonical marine successional trajectories toward *Pseudoalteromonas* dominance while UEG treatment disrupted this progression and sustained significantly greater community evenness across both bacterial and eukaryotic domains. At current settings, the system consumed 127 Wh/day, with energy modeling suggesting meaningful reductions are achievable through irradiance and duty-cycle optimization.

## Highlights

• UEG surfaces maintained <15% fouling coverage over 12 weeks vs. 100% in controls.
• Fouling suppression by UEG persisted and widened throughout the deployment.
• UEG suppressed macrofouling recruitment, sustaining an early-stage biofilm state.
• UEG altered bacterial succession and reduced eukaryotic macrofouling dominance.
• Antifouling effect achieved at a low energy cost of 127 Wh/day.

## 1 Introduction

Marine biofouling imposes substantial operational and economic costs across shipping, aquaculture, and oceanographic sensing, and the dominant mitigation strategies carry environmental costs of their own. Biofouling proceeds through a temporally ordered succession [1], [2] within seconds of immersion, adsorbed organic macromolecules form a conditioning film that primes the surface for attachment [3]. Bacteria, often dominated by Rhodobacterales, are typically the first biological colonizers, producing extracellular polymeric substances (EPS) within hours that anchor cells to the surface and further condition it by altering wettability, charge, and roughness in ways that promote subsequent attachment [4], [5], [6]. Diatoms and other microeukaryotes follow within days, in some cases preceding bacterial attachment depending on environmental conditions; motile diatoms secrete their own carbohydrate-rich EPS for adhesion and gliding motility, while dinoflagellates, fungi, and protozoa instead exploit the existing mucilaginous matrix to attach [4], [7]. These contributions generate the biochemical cues that recruit secondary microbial colonizers and, ultimately, settling invertebrate larvae and algal propagules that form mature macrofouling communities [8], [9]. Because the earliest stages govern the trajectory and rate of the entire sequence, disrupting pioneer colonization before EPS-mediated stabilization is the most effective point for sustained intervention [2], [10].

Ultraviolet-C (UVC) radiation inactivates cells through direct photodamage rather than chemical release [11], [12], [13], [14]. Conventional UVC systems, however, irradiate surfaces externally through the water column, where light is attenuated by absorption and scattering and where surface topography and accumulating biomass protect the biofilm from external stressors [15], [16]. UV-emitting glass (UEG) couples germicidal UV light from edge-mounted LEDs into an optically transparent substrate and scatters it outward through cladding-embedded silica nanoparticles, delivering UVC directly at the solid-liquid interface where pioneer cells attach and before they are shielded within an EPS matrix [11], [17], [18]. By irradiating from the surface outward rather than across the water column, this bottom-up geometry circumvents the attenuation and shadowing that constrain external systems and operates at substantially lower irradiance, reducing potential impact on surrounding ecosystems. We have previously shown that UEG suppresses biofilm formation under controlled laboratory conditions and during short-term marine exposure [11], [17], [18]. These studies established the underlying principle but relied on bench-scale or short-duration configurations that could not be left to operate unattended under field conditions and therefore could not address how the technology behaves over the timescales on which problematic irreversible fouling develops.

Here we report the first fully deployable UEG system: a watertight, continuously operated system intentionally designed for on/off energy cycling, recover energy consumption, and record electrical interruptions. This device enabled the first multi-month marine evaluation of surface-integrated UV antifouling, allowing the evaluation of antifouling performance as fouling pressure intensifies, and communities mature over months rather than days. Additionally, we can begin to understand the mechanism of inhibition from self-emitting surfaces that have led to the high antifouling efficacy reported in earlier work. Prior work on externally applied UVC reported substantial biomass reduction but comparatively stable community composition, suggesting a decoupling between structural disruption and community organization [19]. Because UV-tolerance traits vary widely across taxa [20], [21], sustained interfacial irradiation may instead act as a selective pressure that reshapes which organisms colonize and persist, and whether bottom-up delivery alters the successional trajectory, a question that has not been examined.

In this study, we (1) quantify the long-term antifouling performance of a deployable UEG system over three months of continuous marine submersion, (2) characterize how sustained interfacial UV reshapes biofilm development and microbial community succession relative to non-emitting surfaces, using convergent structural imaging and 16S and 18S rRNA gene amplicon sequencing (hereafter 16S/18S metabarcoding), and (3) evaluate the system’s energy demand to assess scalability for autonomous deployment.

## 2 Methods and materials

### 2.1 UEG and control lens fabrication

UV-emitting glass (UEG) and control lenses (101 mm diameter, 6 mm thickness) were coated on one side with a proprietary nanoparticle composite by Optical Waters Co. (USA). UEG lenses were coated to emit UV light across the surface when connected to the UV LED. Control lenses received the same coating to replicate UEG surface properties, without UV emission. Waterproof UEG housings were fabricated to integrate the UEG lens with six UV-LEDs (nominal 265 nm; peak emission 267 nm, full-width half-maximum 15 nm; 130° lens; 20 × 20 mm Starboard MCPCB; Violumas Inc. Fremont, CA) for marine deployment. The housing was designed to withstand hydrostatic pressure equivalent to a 10 m water depth and was constructed from UV-resistant materials. The enclosure covered 15 mm of the lens perimeter, resulting in an exposed lens area of 56.7 cm^2^. The final enclosure design is shown in Fig. 1a with detailed dimensions, multi-angle views in Fig. S2.

**Fig. 1:**
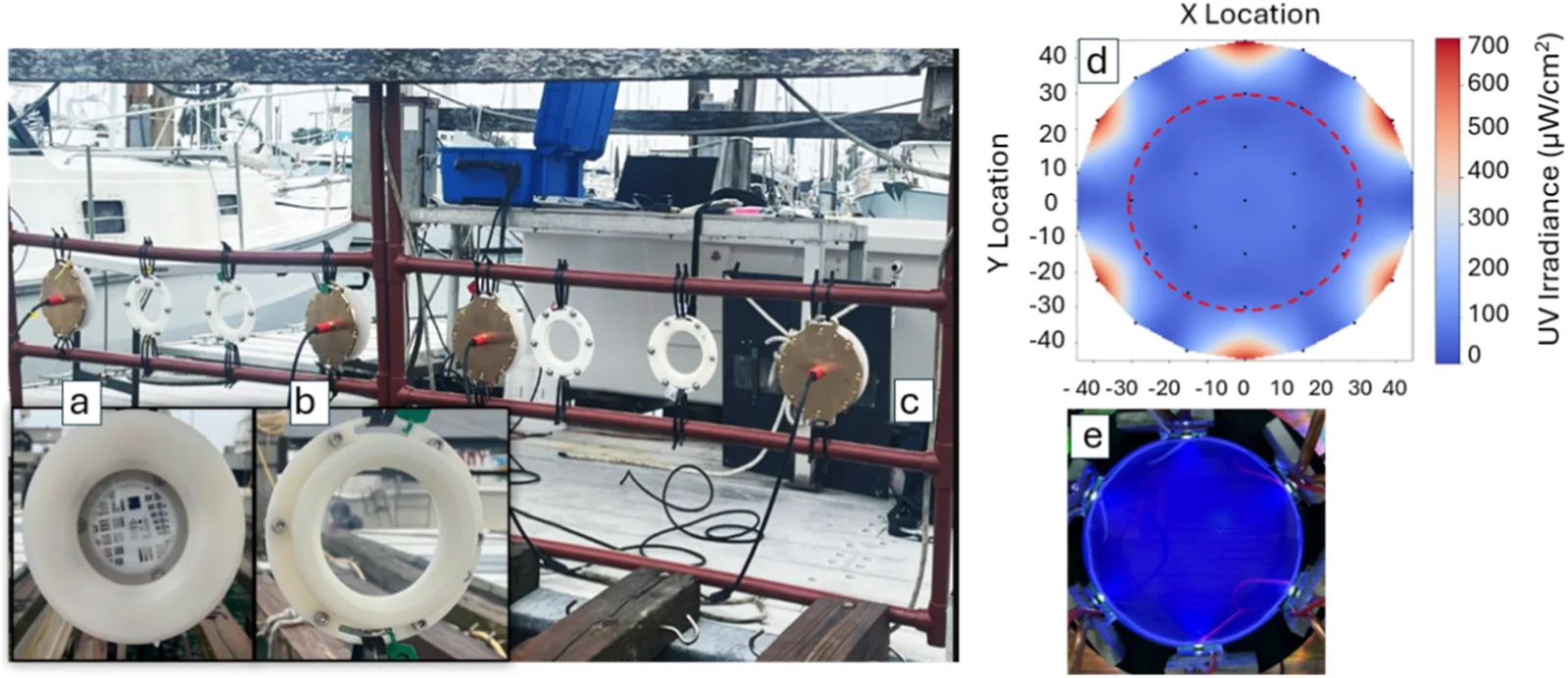
UEG (a) and control (b) lenses housing final design, submersion setup (c), spatial UV irradiance of the UEG surface at 50% input power (d); the red circle delineates the seawater-exposed lens region during submersion testing, where the average UV irradiance was 67 μW/cm^2^, and representative photograph of the UEG surface (e). (For interpretation of the references to color in this figure legend, the reader is referred to the Web version of this article.)

Control lens housings provided mechanical support for the lens without UV-LEDs or electrical connections. Each control housing consisted of two parts that secured the lens in a window-frame configuration and were fastened together with screws (Fig. 1b). The housing geometry matched that of the UEG housing to ensure comparable hydraulic effects and exposed the same coated lens area (56.7 cm^2^) to the surrounding water during submersion (Fig. 1b). Full UEG and control housings and electrical specifications are provided in SI Section 1. Additionally, thermal simulation parameters, and the manufacturer’s 24-h hydrostatic pressure test results are provided in SI Section 2.

### 2.2 UV emission characterization

UV emission from the UEG lenses was characterized through bench-scale spectroradiometry, optical transmittance measurements, and ray-tracing simulations to quantify the irradiance delivered to the lens surface under both ambient and submerged conditions. The UV emission profile of each UEG lens was measured in ambient air using a calibrated spectroradiometer (AvaSpec-2048L, Avantes, Louisville, CO, USA; spectral range 200 to 1100 nm) in a custom 3D-printed nylon (PA2200) lens holder that accommodated the UEG lens and six UV-LEDs arranged hexagonally in direct contact with the lens edge to maximize optical coupling (Fig. S2). The LEDs were mounted on an ice-cooled heat sink with < 1 mm thermal paste layer to minimize temperature drift during measurement. UV spectral irradiance (μW/cm^2^) was measured in duplicate at predefined radial locations across the lens surface using a 5 mm^2^ sensor positioned normal to the lens at a fixed distance of less than 1 mm. The closest measurement point to each UV-LED was located 3 mm from the LED, with subsequent points spaced at 13.6 mm intervals forming concentric rings from the lens perimeter toward the center (see section 3.1). All three UEG lenses were characterized and average values are reported. Transmittance of the coated UEG lens was additionally measured across the 100 to 1100 nm range using a Genesys 10S spectrometer to verify optical properties prior to deployment. Underwater irradiance was estimated through ray-tracing simulations performed by Violumas Inc. (Fermont, CA) and fully described in SI section 3.

### 2.3 Marine deployment

UEG and control housings were mounted on a PVC support frame using heavy-duty zip ties and deployed at Florida Tech’s static immersion test site at Cape Marina, Port Canaveral, FL (Fig. 1c) from February through May 2025 at a fixed water depth of 81 cm (32 inches). In total, 3 UEG and 4 control units were deployed; All submerged units were continuously monitored for weekly data collection, while 1 control and 1 UEG unit were retrieved at months 1, 2, and 3 for destructive analysis. An additional control unit fabricated using the brass-and-PVDF construction of the UEG housing but without UV-LEDs was deployed in parallel to test for substrate-material effects on fouling accumulation and retrieved at month-1. Furthermore, one additional control unit was retrieved after one month solely to evaluate the effect of shipping conditions on fouling integrity during transport; this unit was excluded from subsequent analyses. The electronics control box, containing the LED drivers and timing controllers, was positioned on the test barge and remained out of the water throughout the experiment. Water temperature during the deployment was 16.4 ± 1 °C during weeks 0 to 1 and 22.2 ± 1 °C during weeks 2 to 12; the range of turbidity on site was 1.37 ± 1.4 FNU at submersion depth and salinity was 34 ± 2 PSU. Details of the measurements are included in SI Section 4. The LEDs were operated at 50% of maximum power under a cyclic regime of 24 on/off cycles per day (30 min on/30 min off). Details are provided in SI section 5.

### 2.4 Sample retrieval and characterization

Weekly in situ monitoring and monthly destructive retrieval were conducted throughout the three-month deployment. In situ weekly monitoring of each UEG assembly included recording of electrical performance parameters (voltage [V], current [A], power [W], and cumulative energy consumption [Wh]) from the LED driver, coupled to each unit. Prior to lifting each unit from the water, LED drivers were disconnected to mitigate thermal stress to the light-emitting components and human exposure to UV light. To reduce edge effects during subsequent fouling analyses, a non-shedding swab was used to clean an approximately 5 mm perimeter region surrounding the lens while avoiding direct contact with the lens surface. For control samples, the uncoated side of the lens was gently wiped during weekly monitoring and prior to in-lab analyses with a Kimwipe to prevent interference with fouling coverage assessment of the coated surface. Weekly visits also documented any animal or human interference, electrical disturbances, significant weather events, and water temperature variations. During Week 5, the LEDs in one UEG unit failed and were replaced over a two-day period; the affected UEG lens and its corresponding control were refrigerated at 4 °C and re-immersed once LED replacement was complete. Observations of energy delivery interruptions during the deployment are reported in Section 3.2. Each month, one UEG housing and one control housing were retrieved from the deployment site and transported to the laboratory for post-exposure characterization. Details on shipping conditions are provided in SI section 6.

All fouling coverage, OCT, CFU, microscopy, and sequencing analyses were performed exclusively on the coated side of both UEG and control lenses.

### 2.5 Fouling and biofilm analysis

Retrieved lenses were characterized using four complementary approaches: visual fouling coverage assessment, optical coherence tomography (OCT), colony-forming unit (CFU) enumeration, and light microscopy. Each lens was photographed immediately after retrieval and visual fouling coverage was estimated following ASTM D6990 by dividing each image into a grid and quantifying the fraction of grid sections exhibiting visible biofilm growth (grid size was 0.5 cm^2^, and number of grid cells was 227). Image division and scoring were performed manually.

OCT imaging was used to obtain two- and three-dimensional biofilm images and to quantify fouling thickness using a Ganymede Series spectral-domain OCT system (Thorlabs) with a nominal center wavelength of 905 nm. Prior to imaging, lenses were resubmerged in phosphate-buffered saline (PBS). Imaging was conducted under submerged conditions to maintain the fouling in a fully hydrated state and to avoid mixed air-water optical interfaces, which would introduce refractive index heterogeneity and compromise thickness measurements. The instrument refractive index was set to 1.33. Mean biofilm thickness was determined through manual segmentation from 2D OCT images by measuring the distance between the upper and lower biofilm interfaces at 20 locations with biofilm coverage. These 20 locations represent technical replicates characterizing thickness variability within a single lens; biological replication was n = 1 lens per condition (UEG or control) per timepoint. The lower boundary was defined as the substrate-biofilm interface, identified by the hyper-reflective signal corresponding to the underlying surface. The upper boundary was defined at the biofilm-liquid interface using manual intensity thresholding, in which low-intensity dark regions were interpreted as the surrounding aqueous medium, and higher-intensity colored regions were identified as light-scattering biofilm biomass. Segmentation and measurements were performed by a single observer. Fouling volume was calculated from the coverage and thickness data.

For CFU analysis, fouling material was removed from each lens surface using a sterile cell scraper and suspended in 10 mL of PBS. Fouling was removed from each lens surface using a sterile cell scraper with a 20 mm pivoting blade. The lens surface was scraped systematically in a top-to-bottom motion, with each 20 mm-wide strip scraped 10 times to ensure complete removal of surface biomass before proceeding to the adjacent strip. This scraping procedure was applied identically to UEG and control lenses to standardize biomass recovery across sample types. Suspensions from control lenses were vortexed for 2 min to promote detachment from larger fouling aggregates, while UEG suspensions, which contained no hard fouling, were vortexed for 1 min to ensure homogeneity, consistent with reports that mature, matrix-embedded biofilms require greater disaggregation effort than simple planktonic suspensions [22]. This disaggregation protocol was not independently validated in the present study. Following vertexing, suspensions were allowed to settle for 1 min to allow large particulate debris and fouling fragments to settle prior to pipetting, preventing clogging of the pipette tip. The supernatant was then serially diluted and plated in duplicate on Marine Agar (Fisher Scientific, 2216). The settled fraction was not independently evaluated. Given the brief settling time and its intent to remove only larger debris, any effect on microbial recovery is expected to be minimal. Duplicate plates represent technical replicates from the same dilution of a single lens’s suspension; biological replication was n = 1 lens per condition per timepoint. Plates were incubated for 24 h at 37 ± 1 °C, and CFU per cm^2^ was recorded. CFU/cm^2^ was calculated as:

(1) $${CFU/cm}^{2}=\frac{\left(N\times D\times V_{s}\right)}{\left(V_{p}\times A\right)}$$

where, *N* is the number of colonies counted on a plate, *D* is the reciprocal of the dilution plated, V_s_ is the total volume of the resuspended fouling sample (10 mL), *V*_*p*_ is the volume of diluted suspension plated, and *A* is the sampled surface area of the lens (56.7 cm^2^).

CFU counts on Marine Agar capture only viable, culturable bacteria, excluding non-culturable bacteria and eukaryotic microorganisms, so CFU values and the resulting LRVs do not represent total bacterial abundance. The wider fouling community was therefore assessed independently using OCT imaging, microscopy, and 16S and 18S rRNA gene metabarcoding.

Fouling coverage scoring and OCT segmentation were each performed by a single observer, without an inter-rater reliability assessment. Blinding was not feasible because fouling differed visibly between UEG and control lenses at every timepoint. Given the size of these differences, observer judgment is unlikely to have changed the direction of the treatment effects, although it may have influenced individual estimates. Independent scoring by multiple observers should be used in future studies to reduce observer-dependent variability. Light microscopy was used to image biofilm structure and identify representative fouling microorganisms. Briefly, 5 μL of the resuspended fouling sample, prepared from the same stock suspension used for CFU enumeration, was pipetted onto a glass microscope slide and covered with a coverslip. Brightfield images were acquired using an AMG EVOS FL microscope, with imaging fields selected from regions containing visible microbial aggregates.

### 2.6 16S and 18S metabarcoding and analysis

DNA isolation, library preparation, and sequencing followed Alidokht et al., [11]. Briefly, DNA was extracted from biofilm material recovered from each lens using a DNeasy PowerSoil Pro Kit (Qiagen, Hilden, Germany) following the manufacturer’s instructions. DNA concentrations were measured with a Qubit Fluorometer using the Qubit dsDNA HS Assay (1:10 dilution), and DNA samples were stored at −80 °C until library construction. Amplicon libraries were prepared and sequenced by Novogene Co. Ltd. on an Illumina NovaSeq 6000 platform.

Bacterial community composition was characterized by amplifying the V4 region of the 16S rRNA gene using primers 515F (5′-GTGCCAGCMGCCGCGGTAA-3′) and 806R (5′-GACTACHVGGGTATCTAATCC-3′). Eukaryotic community composition was characterized by amplifying the V9 region of the 18S rRNA gene using primers 1391F (5′-GTACACACCGCCCGTC-3′) and EukBr (5′-TGATCCTTCTGCAGGTTCACCTAC-3′) [23], [24].

Amplicon sequence data were processed with DADA2, including quality filtering, denoising, inference of exact amplicon sequence variants (ASVs), paired-end read merging, and chimera removal. Taxonomic assignment of 16S ASVs was performed using the SILVA rRNA reference database (version 138.2), and 18S ASVs were annotated against the Protist Ribosomal Reference (PR2) database (version 5.1.1) [25], [26]. All downstream community analyses were conducted in R (version 4.2) using established microbiome analysis packages [27], [28]. Alpha diversity within each sample was characterized using Chao1 richness, Shannon diversity, and inverse Simpson indices. Beta diversity was assessed using Bray-Curtis dissimilarity and visualized using non-metric multidimensional scaling (NMDS) ordination. Community overlap between treatments and timepoints was summarized using Venn diagrams constructed from presence-absence data to identify shared and unique taxa.

### 2.7 Statistical analysis

Measurements are reported as mean ± standard deviation across within-sample technical replicates: biofilm thickness was averaged across 20 locations per lens by OCT, and CFU counts were averaged across two technical plating replicates. LRV were calculated as log_10_ (CFU_control_/CFU_UEG_) for each monthly sampling pair. For the 12-week continuous monitoring, mean weekly coverage and cumulative energy consumption are reported across UEG and control units (n = 3, 2, 1 for weeks 1-4, 5-8, 9-12 respectively). Statistical analyses were performed to evaluate differences in fouling coverage among replicate lenses. Paired t-tests were conducted to assess differences between replicate units for periods in which replicate measurements were available, with statistical significance defined at p < 0.05. For the monthly destructive sampling, n = 1 UEG and 1 control unit were recovered per timepoint. Statistical contrasts of alpha diversity and community composition between treatments and across timepoints cannot be discussed with statistical significance under the n = 1 design; microbial community results are therefore reported and interpreted descriptively.

## 3 Results and discussion

### 3.1 Device characterization and UV delivery to submerged surfaces

**Thermal stability under continuous submerged operation:** Steady-state thermal simulations confirmed that LED operation does not approach thermal failure limits during continuous submersion. Depending on thermal paste thickness (0.1-0.5 mm), LED board temperatures ranged from 38.6 to 43.6 °C, substantially below the 120 °C maximum junction temperature specified for the Violumas UV-LEDs used in this work (thermal resistance approximately 30 °C/W), beyond which performance degradation, wavelength shifts, accelerated aging, encapsulation softening, and permanent failure can occur [29], [30]. To minimize thermal stress during long-term deployment, the assembled UEGs received a thin thermal paste layer (less than 0.5 mm) applied using a pressure-spreading method. Full thermal simulation outputs at both 0.5 mm and 0.1 mm paste thicknesses are provided in Fig. S3.

**UV irradiance delivered to the lens surface:** Spatial UV irradiance at the UEG lens surface was characterized to evaluate the irradiance delivered across the seawater-exposed area and to determine the operating conditions used for the submersion experiments. Measurements were conducted at input power levels of 100%, 50%, and 30%, with sampling locations arranged in concentric circular patterns around the LED ring, as illustrated in Fig. S5. Overall, average UV irradiance values within the seawater-exposed regions were 115, 67, and 40 μW/cm^2^ at 100%, 50%, and 30% input power, respectively. A proportional decrease in irradiance was observed with decreasing power input, while the spatial distribution of UV light across the lens surface remained generally consistent across all operating conditions.

Based on these measurements, a 50% power input setting was selected for the deployment experiments. At this operating condition, the measured irradiance across the water-exposed surface ranged from 147 to 51 μW/cm^2^ (Fig. 1d–c). Simulation results further indicated that the effective surface irradiance was reduced by absorption within the housing material and seawater by approximately 4% and 14%, respectively, resulting in a final delivered irradiance of approximately 55 μW/cm^2^ at the surface when submerged. This irradiance is comparable to that reported by Torkzadeh and Cates [14] and Naik et al. [19] (60 μW/cm^2^). Under the operational cycling conditions described, the delivered daily fluence was 2376 mJ/cm^2^/day, within the range previously associated with disruption of bacterial surface attachment and inhibition of early-stage biofilm formation [11], [17]. This irradiance was therefore used as the operational reference condition for the deployment experiments described in Section 3.2.

**Selection of the operational duty cycle.** Preliminary tests across continuous and intermittent UV exposure regimes showed that 12-h daily protocols of repeated short cycles substantially outperformed long single cycles in biofilm control, consistent with UVC being most effective against cells at or before attachment, when they remain unprotected by extracellular polymeric substances [11], [31]. Full cycling test data and details of the test system are provided in SI Section 5.

### 3.2 Sustained low fouling coverage over multi-month marine deployment

**Weekly coverage and visual evidence.** Fig. 2 illustrates the weekly monitored data where UEGs held fouling coverage below 15% across the entire 12-week deployment, while controls progressed to 100% surface coverage within 2 weeks. No statistically significant differences in fouling coverage were observed among replicate UEG or replicate control units (p > 0.05). The 100% fouling coverage observed on the controls after two weeks is consistent with the broader literature on unprotected surfaces in marine environments [32]. Diatoms and bacteria colonize glass surfaces within hours to days of ocean exposure [33], with significant biofilm formation documented on glass after as few as 10 days even at 4700 m depth [34]. Full or near-full fouling coverage on unprotected surfaces within comparable timescales has been reported across multiple field studies, including 100% macrofouling on stainless steel, titanium alloy, and PVC panels after 3 months of seawater immersion [35], 94.7% coverage on pontoon surfaces within 2 months [36], and 97–100% microbial coverage on seafloor-deployed plates after 3 months [37].

**Fig. 2:**
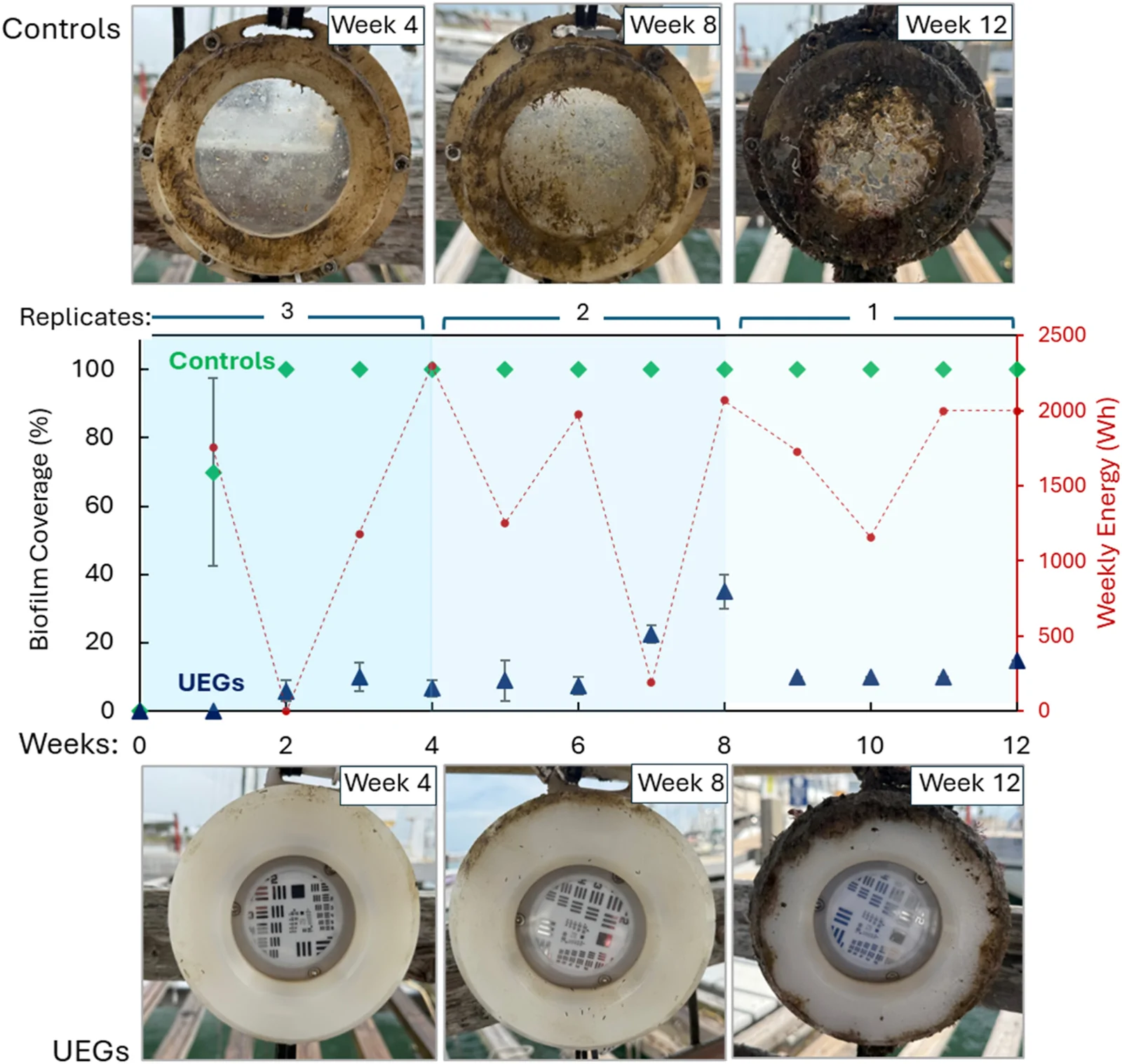
Fouling coverage and weekly UV energy delivery over 12 weeks of marine deployment. Representative images of control and UEG samples at weeks 4, 8, and 12 are shown, with a USAF resolution target used to indicate optical clarity. Full weekly image sets are provided in Fig. S7. Fouling coverage is plotted for UEG (blue) and control (green) units, alongside weekly UV energy delivery (red). Error bars represent varying replicate numbers across time (n = 3 for weeks 1-4, n = 2 for weeks 5-8, n = 1 for weeks 9-12). (For interpretation of the references to color in this figure legend, the reader is referred to the Web version of this article.)

The visual progression of fouling in Fig. 2 supports the coverage measurements. Control surfaces transitioned from clear at week 1 to thin biofilm by week 2, with hard fouling appearing thereafter and progressive accumulation through week 12. UEG surfaces remained substantially clean throughout, with the USAF resolution target visible behind the UEG glass providing an independent visual indicator of optical clarity across the deployment.

**Energy delivery corresponded with performance**. Brief increases in UEG fouling coverage during weeks 3 and 7-8 (Fig. 2) coincided with unintended reductions in UV energy delivery caused by power interruptions. Weekly energy consumption dropped to near zero in week 2 and to approximately 190 Wh in week 7. Fouling coverage increased slightly in week 3 following the first interruption and more noticeably in weeks 7-8 following the second, returning to baseline once operation resumed. This response highlights the importance of continuous UVC irradiation during early biofilm development, when microbial cells are most susceptible to photodamage and loss of replication capacity [38], [39], [40]. Temporary interruptions allow microorganisms to colonize conditioning layers and establish protected microenvironments that reduce subsequent UV effectiveness through shielding and localized regrowth [41]. Continuous UV operation has similarly been shown to maintain fouling below 5% over a 2-month marine trial [42], while declining UV-LED output over extended operation allowed fouling re-establishment on previously protected surfaces reinforcing that maintained UV delivery, governs long-term performance [43].

**Quantitative validation through destructive sampling:** Independent destructive sampling at months 1, 2, and 3 (n = 1 UEG and 1 control per timepoint, with each timepoint deployed as a separate sample set) provides quantitative and structural support for the coverage data. OCT derived fouling volume and thickness, and CFU values are reported as mean ± SD across technical replicates (20 OCT locations per lens; duplicate CFU plates per lens) within each single lens, and do not reflect biological replication. Control fouling volume increased progressively from 15 ± 2 mm^3^ at month-1 to 54 ± 2 mm^3^ at month-3, and OCT-derived fouling thickness rose from 0.3 ± 0.4 mm to 1.0 ± 0.5 mm over the same period (Fig. 3). By contrast, UEG samples showed substantially lower fouling, with volume and thickness increasing only marginally from 0.047 ± 0.003 mm^3^ and 0.008 ± 0.005 mm at month-1 to 0.89 ± 0.07 mm^3^ and 0.10 ± 0.06 mm at month-3. UEG month-2 volume and thickness data were not available due to coating detachment during ex-situ imaging. CFU enumeration yielded LRV of 2.3, 0.46, and 3.1 for months 1, 2, and 3, respectively. These values are consistent with or exceed those reported in the prior UEG short-term marine deployment (LRV 1.79 over 20 days) [17] and laboratory-based UEG studies (LRV > 3 at 265 nm; LRV > 2 under optimized conditions) [11], [18], and fall within the range reported for external UVC surface irradiation systems, where biofilm-bound bacterial inactivation typically yields LRVs of 1.5-2.5 depending on fluence, wavelength, and biofilm maturity [13], [14]. The relatively higher LRVs observed in the present study may be attributed in part to the higher and spatially uniform UV irradiance delivered directly from the UEG surface, which minimizes shadowing effects and ensures consistent fluence delivery across the illuminated area. The month-2 LRV of 0.46 coincides with the energy delivery interruption identified in the continuous monitoring data, reinforcing the UV delivery-dependence of antifouling performance rather than indicating an inherent limitation of UEG operation. Fig. 3 (right) illustrates OCT images of biofouling on control and UEG at month-3 along with corresponding microscopy images. OCT surface topology and microscopy analyses at month-3 revealed the development of thick and structurally heterogeneous fouling layers on the control surfaces. These communities included macrofouling organisms such as calcareous tubeworms, encrusting bryozoans, and barnacles, together with dense biofilm aggregates containing eukaryotic cells embedded within EPS and associated organic material. Fig. S8 further illustrates the temporal progression and percent surface coverage of fouling organisms on both control and UEG surfaces throughout the submersion period. In contrast, UEG surfaces exhibited substantially thinner biofilm accumulation, characterized by patchy and spatially dispersed deposits rather than the continuous multilayer fouling observed on the controls. The ∼15% fouling coverage on UEG surfaces after three months may reflect the interaction between the UV duty cycle and continuous submersion in marine. Recurring off-cycles provide windows for initial cell attachment without inactivation pressure; once an attached layer is established at a site, it can attenuate outgoing UVC fluence [13], reducing exposure to cells attaching at the outer, water-facing surface and permitting continued outward accumulation during both off- and on-cycles. This mechanism is consistent with the reduced inactivation observed during the month-2 UV delivery interruption and the persistent, patchy coverage at week 12, suggesting fouling suppression depends on both duty cycle and biomass-driven fluence attenuation once colonization begins. Confocal microscopy with depth-resolved live/dead staining, combined with spatially resolved fluence measurements through developing biofilm, would directly test this mechanism and is a priority for future work. Separately, microscopy and photographic observations further indicated reduced settlement of Chelonibia spp. (barnacles), tubeworm-associated taxa, and bryozoans on the UEG surfaces. Reduced bacterial colonization on UEG surfaces likely limited secondary colonization through several interrelated mechanisms. Bacterial density and community composition are established determinants of invertebrate larval settlement [44], [45], so a thinner bacterial biofilm would provide weaker density-dependent settlement cues. Bacteria and their EPS generate water-soluble and surface-bound chemical signals, including quorum-sensing molecules, that larvae and propagules use to select settlement sites [44]. Lower bacterial biomass would diminish these signals as well as the attachment substrate and nutritional resources available to grazers and subsequent colonizers. The reduced settlement of macrofouling observed on UEG surfaces is consistent with previous studies demonstrating that UV-based antifouling strategies suppress macrofouling recruitment and maintain surfaces in an early-stage biofilm state unfavorable for larval settlement and subsequent community development [19], [42], [46]. A substrate control comparing the nylon control housing and the white plastic UEG housing showed no significant difference in fouling accumulation (Fig. 3), confirming that the observed difference reflects UV treatment rather than case material.

**Fig. 3:**
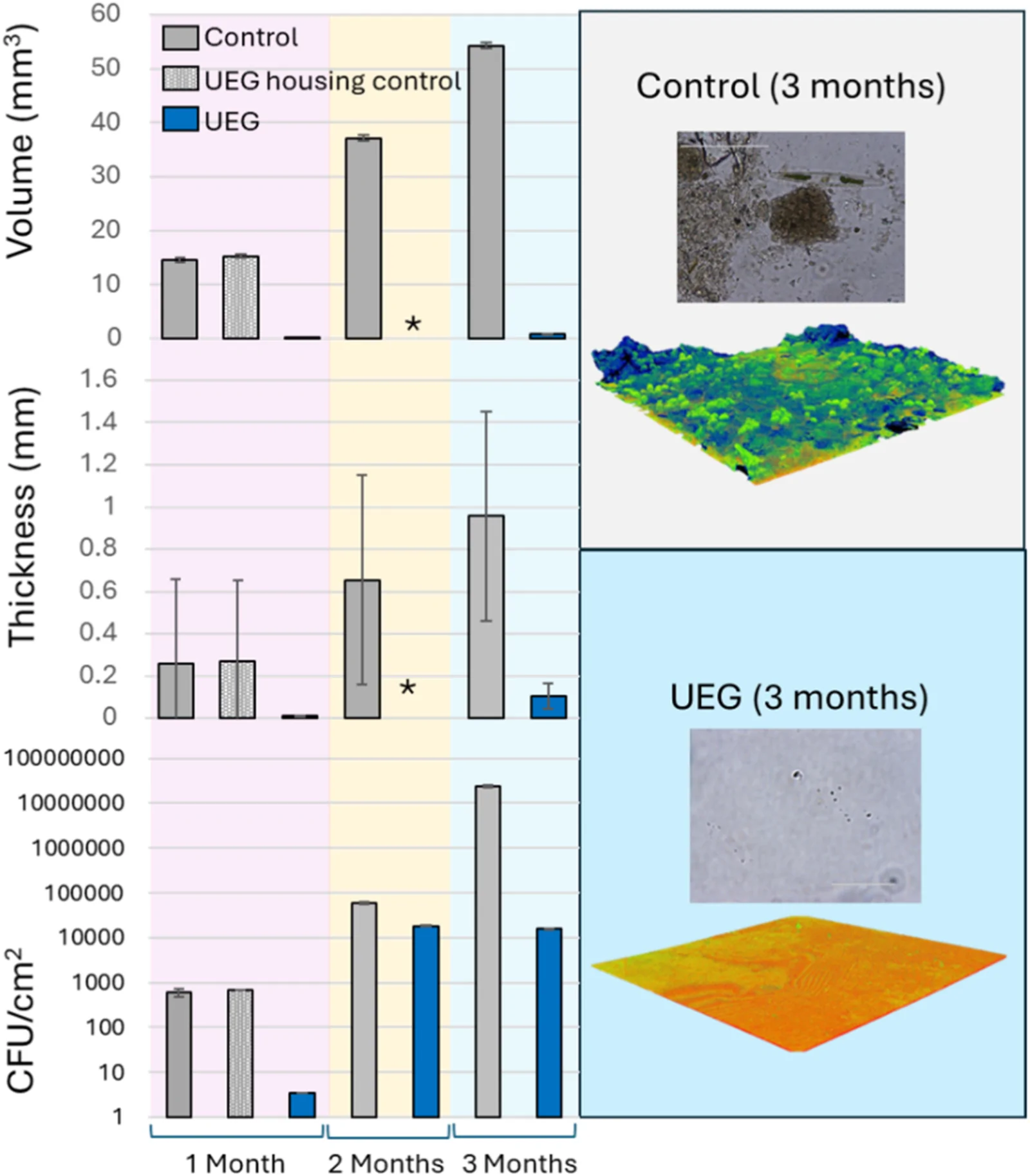
Quantitative fouling analysis of UEG and control samples over three months of submersion. The left panel presents fouling metrics, with month-2 UEG volume and thickness data unavailable due to coating detachment during ex-situ OCT imaging (*). Error bars represent standard deviation across replicate measurements from a single lens. The right panel shows representative OCT surface topography (scanning area 4.5 mm × 4.5 mm) and microscopy images (100 μm scale bar) after three months, with full replicate datasets provided in Figs. S9 and S10.

### 3.3 Microbial community composition differs between UEG and control surfaces, with fewer macrofouling-associated taxa on UEG

16S metabarcoding of monthly samples (n = 1 per condition per month) suggested that bacterial succession may have followed a different trajectory under UEG over the three-month deployment. Taxonomic composition differed between treatments (Fig. 4a). Control communities showed a temporal sequence consistent with typical marine biofilm succession. Month-1 communities transitioned from early colonizers at Month-1 (*Erythrobacter* 12.3%), with *Psychrobacter*, *Altererythrobacter*, *Winogradskyella,* and an unclassified Rhodobacteraceae genus also prominent, consistent with Rhodobacteraceae as primary colonizers of submerged surfaces in coastal waters [4]. By Month-2, controls shifted to a *Vibrio*-dominated assemblage (44.4% Vibrio, 22.7% *Aestuariibacter*). *Vibrio* species are capable biofilm formers that can exploit successional niches and contribute to matrix development through quorum sensing [4]. At Month-3, controls were dominated by *Pseudoalteromonas* (70.9%), consistent with the competitive success of this genus on marine surfaces, where secondary metabolite production can inhibit or displace other taxa [47]. The presence of *Alteromonas* and *Marinobacter* at Month-3 is also consistent with conventional marine succession, in which early colonizers are replaced by a later-stage community shaped by competition [48].

**Fig. 4:**
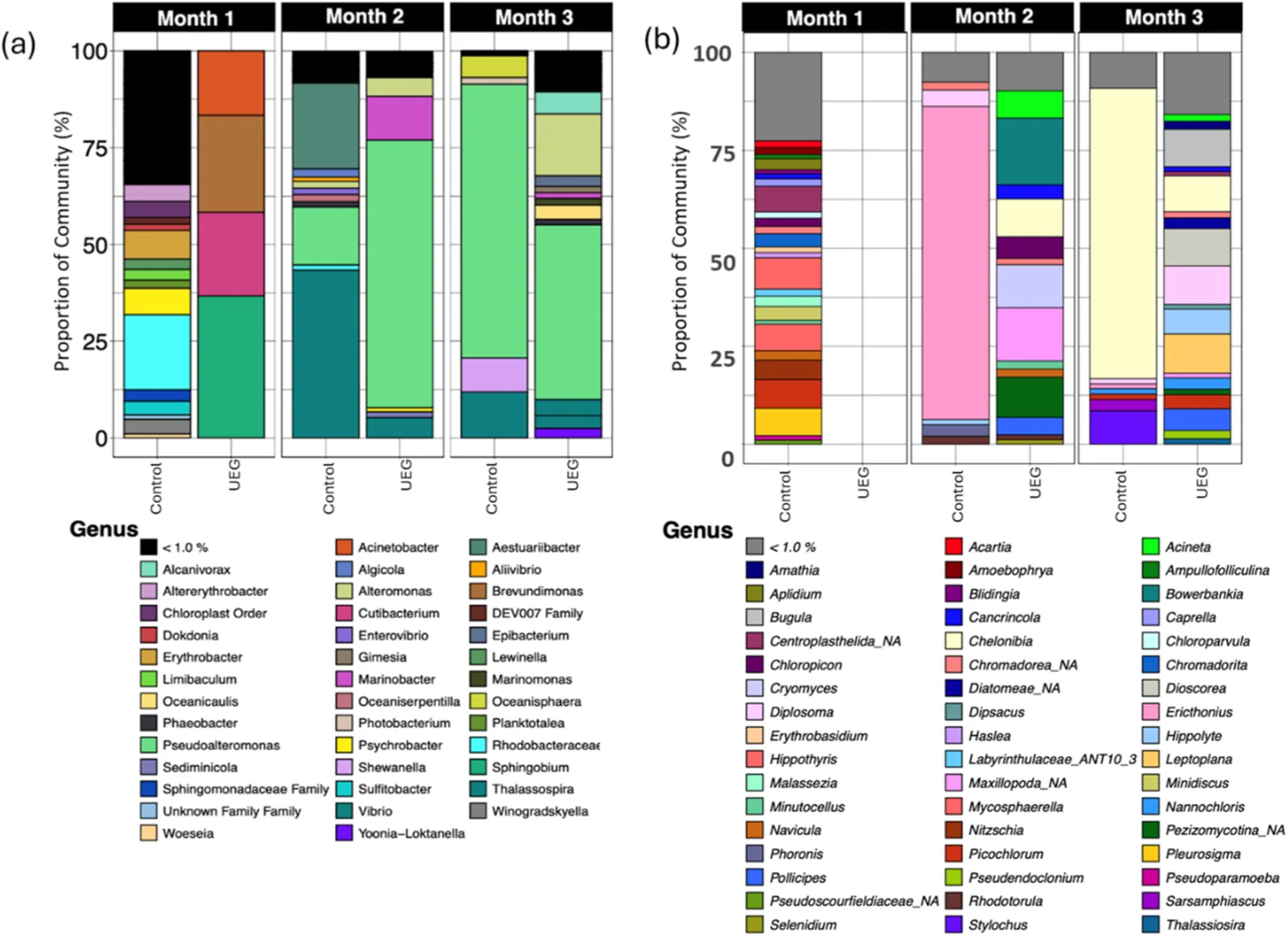
Microbial community composition on control and UEG surfaces across the three-month deployment. Relative abundance of bacterial genera based on (a) 16S rRNA and (b) 18S metabarcoding. Less abundant taxa (<1.0%) are pooled into “Other.” The month-1 UEG eukaryotic sample failed to sequence due to low biomass and is not shown in panel B.

Succession on UEG surfaces may also have been delayed rather than disrupted, with communities at each timepoint resembling earlier stages of the control trajectory. Month-1 UEG communities (*Sphingobium, Brevundimonas, Cutibacterium, Acinetobacter*) did not closely resemble the Month-1 control community, which were dominated by *Rhodobacteraceae*-associated colonizers (*Erythrobacter, Psychrobacter, Altererythrobacter, Winogradskyella*). Similarly, the Month-3 UEG assemblage (*Pseudoalteromonas* 46.1%, with *Alteromonas*, *Alcanivorax*, *Thalassospira*, *Oceanicaulis*, and Vibrio) differs in composition from the *Rhodobacteraceae*-dominated Month-1 control community, though both share a comparatively even, non-dominant structure relative to the *Pseudoalteromonas*-dominated Month-3 control. With a single sample per timepoint, a delayed-succession model cannot be excluded, and both interpretations remain possible.

UEG samples showed a different temporal pattern. Month-1 communities contained only four detected genera, *Sphingobium, Brevundimonas, Cutibacterium*, and *Acinetobacter*. *Pseudoalteromonas* became dominant at Month-2 (69.6%), but decreased by Month-3 to 46.1%, while *Alteromonas, Alcanivorax*, *Thalassospira*, *Oceanicaulis*, and *Vibrio* increased. The Month-3 UEG sample did not show the near-dominance by *Pseudoalteromonas* observed in the Month-3 control and instead contained a more even distribution of multiple taxa. This pattern is consistent with either a disrupted or a delayed successional trajectory relative to controls. *Sphingobium* and *Brevundimonas*, both common in oligotrophic seawater and surface-attached assemblages [49], [50], [51], [52], appeared more prominently under UEG treatment, suggesting either selection for taxa adapted to altered surface conditions or reduced dominance by strong competitors like *Pseudoalteromonas*. *Cutibacterium*, is a common skin-associated genus and a well-documented contaminant in low-biomass 16S rRNA gene sequencing [53]. It was detected only in the Month-1 UEG sample, which had the lowest biomass in the bacterial dataset. A PBS extraction blank processed with the samples did not pass pre-sequencing QC due to insufficient DNA and was not sequenced; therefore, we could not determine whether *Cutibacterium* was introduced during field sampling and transport or during DNA extraction and molecular processing.

**Eukaryotic communities show reduced macrofouling dominance on UEG surfaces.** 18S metabarcoding characterized eukaryotic biofilm composition across the three-month deployment (n = 1 per condition per month). The Month-1 UEG sample failed to sequence due to low yield and was excluded. Control communities exhibited pronounced temporal turnover (Fig. 4b). Month-1 controls were diverse, with 27 genera above 1% relative abundance and no single taxon exceeding 10%; the most abundant were *Hippothyris* (8.0%), *Picochlorum* (7.4%), *Pleurosigma* (7.0%), *Mycosphaerella* (6.8%), and an unclassified Centroplasthelida genus. By month-2, control communities became dominated by *Ericthonius* (80.0%), with *Diplosoma* (4.2%), *Phoronis* (2.9%), an unclassified Chromadorea (2.0%), *Rhodotorula* (2.0%), and *Hippolyte* (1.3%) comprising most of the remainder. By month-3, dominance shifted to *Chelonibia* (74.1%), followed by *Stylochus* (8.5%), *Sarsamphiascus* (2.9%), *Nannochloris* (1.4%), *Picochlorum* (1.4%), *Diplosoma* (1.3%), and *Ericthonius* (1.2%). The shift from *Ericthonius* at month-2 to *Chelonibia* and other fouling-associated taxa at month-3, together with earlier detection of diatom and crustacean-associated sequences, indicates temporal turnover toward macrofouling on untreated surfaces.

UEG eukaryotic communities were more even (Fig. 4b). At month-2, the most abundant taxa were *Bowerbankia* (17.0%), an unclassified Maxillopoda (13.6%), *Cryomyces* (11.0%), an unclassified Pezizomycotina (10.3%), and *Chelonibia* (9.6%), with additional contributions from *Acineta*, *Chloropicon*, *Pollicipes*, *Cancrincola*, *Navicula*, *Minutocellus*, an unclassified Chromadorea, *Rhodotorula*, and *Selenidium* each at less than 7%. By month-3, 20 genera exceeded 1% relative abundance, led by *Leptoplana* (10.0%), *Diplosoma* (9.8%), *Bugula* (9.5%), *Dioscorea* (9.5%), *Chelonibia* (9.1%), *Hippolyte* (6.3%), and *Pollicipes* (5.5%). These taxa accounted for approximately 60% of the total community, compared to the seven taxa that accounted for over 90% on controls at the same timepoint. Overall, these 18S rRNA data, although not replicated at each time period, provide a descriptive view of temporal change in eukaryotic biofilm communities. They are consistent with lower dominance by individual taxa and greater evenness on UEG surfaces at later timepoints, which may reflect less established macrofouling; replicated sampling would be needed to test this.

Supplemental diversity analyses were consistent with these compositional patterns (Figs. S11–S13). Alpha diversity, Venn analysis, and Bray-Curtis NMDS ordination showed that UEG communities were generally less diverse but more even and compositionally tighter than controls, while untreated surfaces contained a larger pool of unique bacterial and eukaryotic genera, consistent with broader and more variable colonization.

**Synergies between methods and possible mechanisms of disruption.** The OCT and microscopy structural observations (Figs. S9 and S10) and the 16S and 18S sequencing data (Fig. 4) provide independent but convergent evidence of treatment-specific differences in biofilm development across the three-month deployment. OCT revealed thicker, more heterogeneous fouling layers on controls than on UEGs at each timepoint, with control surfaces accumulating macrofouling organisms (calcareous tubeworms, encrusting bryozoans, and barnacles) and dense biofilm clumps containing eukaryotic cells entrained in EPS and other organic material by month Maxillopoda barnacles, and bryozoan settlers) and detected them at lower relative abundance on UEG surfaces. The concordance among structural, visual, and compositional methods strengthens confidence that the observed patterns are not artifacts of any single approach and provides complementary lines of evidence that help mitigate the limitations imposed by the lack of biological replication (n = 1).

Control surfaces followed a successional trajectory consistent with documented marine biofilm development on inert substrates: conditioning layer establishment, primary bacterial colonization (*Rhodobacteraceae* and other early colonizers at month-1), microeukaryote settlement, and eventual macrofouling establishment [54], [55]. On controls, this trajectory was visible across all three methods, with bacterial succession converging on *Pseudoalteromonas* dominance and eukaryotic succession converging on macrofouling settlers (*Ericthonius* at month-2, *Chelonibia* at month-3). UEG samples diverged from this trajectory at the bacterial stage, with reduced *Pseudoalteromonas* dominance and greater taxonomic evenness, and showed correspondingly reduced macrofouling dominance at the eukaryotic stage. The cross-stage parallel is consistent with disruption at the bacterial stage propagating forward through the successional sequence, though UV may also affect bacterial and eukaryotic colonizers independently.

Beyond the biofilm-mediated effects discussed above, UVC may directly suppress macrofouling by impairing larval viability and recruitment. Direct toxicity to invertebrate recruits has also been implicated in the suppression of macrofouling following UVC treatment [56]. Perepelizin and Boltovskoy [57] reported complete mortality of golden mussel (*Limnoperna fortunei*) larvae following single exposures to 254 nm UVC at fluences of 103-149 mJ cm^−2^, depending on temperature. The daily fluence delivered by UEG (2376 mJ cm^−2^ day^−1^) makes such direct effects plausible, although the fluence received by individual larvae and spores, and thus its sufficiency, could not be determined. Bacterial biofilms, through their role in supporting settlement, provide a plausible indirect pathway through which UVC could also limit macrofouling. Although existing studies support both mechanisms, to our knowledge, no study has directly compared their relative contributions. Neither pathway can therefore be identified as dominant in the present study, and the reduced eukaryotic representation on UEG-treated surfaces may reflect direct UVC effects, biofilm-mediated effects, or a combination of both. Controlled settlement assays will be required to distinguish their contributions. The bacterial divergence under UEG treatment parallels findings by Naik et al. [19] who documented temporal changes in temperate bacterial communities under in-water UVC exposure on both new and older biofilms. Prior UVC studies of marine biofilms have also reported shifts in both composition and abundance across bacterial and diatom assemblages [58], [59], and UV-emitting surfaces have been shown to modify early biofilm development in ways that disrupt the successional stages of biofouling progression[56]. The compounding of differences across successional stages is consistent with the substantial suppression of macrofouling coverage and biomass observed in Section 3.2.

Future work with replicated UEG and control surfaces per timepoint, ideally with statistical comparison of community composition, is needed to confirm the community-level effects suggested here.

In this study, UEG was evaluated at a single deployment depth (81 cm), whereas the chemical, biological, and hydrodynamic conditions of seawater change with depth. Solar UV and visible light are attenuated through absorption and scattering by water, dissolved organic matter, and suspended particles [60], and this attenuation shapes the water column, as dissolved organic matter and phytoplankton generally decrease with available light, shifting communities toward fewer photosynthetic organisms and relatively more heterotrophic bacteria at greater depths. Hydrodynamic conditions also vary with depth and further influence the composition, diversity, and biomass of fouling communities [61]. Because UEG delivers UV directly at the glass-biofilm interface rather than through the water column, its delivered dose should be independent of depth. However, it remains unknown whether depth-related differences in pioneer communities and hydrodynamics affect UEG performance, including the successional dynamics discussed earlier. Replicated deployments along a depth gradient, or across sites with different depths and water conditions, would be needed to confirm its performance at other depths.

### 3.4 Implications for energy efficient deployment

Long-term, low-maintenance deployment of UV-based antifouling systems requires energy consumption to be compatible with available power sources, particularly for autonomous marine sensors and offshore infrastructure where grid power is unavailable. We previously developed a framework for calculating and minimizing the energy budget associated with UV-emitting surfaces [11]. Under the operating conditions used in this study (55 μW/cm^2^ average effective irradiance and a 30 min on/30 min off duty cycle), the energy budget was 127 Wh/day (Fig. S14). Fig. S14 illustrates energy reductions accessible through lower irradiance or reduced fractional on-time. For example, decreasing the irradiance to 10 μW/cm^2^ reduces the projected energy budget to 25.4 Wh/day at 50% fractional on-time and 5 Wh/day at 10% fractional on-time, representing approximately 5-fold and 25-fold reductions relative to the present operating point. A 5-25 Wh/day energy requirement is well within the energy envelope of compact solar- or battery-powered autonomous marine platforms, including solar-powered AUVs (300-900 Wh/day available) and wave-propelled uncrewed surface vehicles with 175-440 W solar arrays [62], [63], supporting integration of UEG antifouling technology into autonomous deployments without dedicated power infrastructure.

The feasibility of these reductions is constrained by UV dose-response biology. Our prior work has shown that UEGs can effectively control biofilm at continuous irradiances as low as approximately 11 μW/cm^2^ over 20 days of marine submersion [17], and Whitworth et al. [64] reported an effective threshold near 1 μW/cm^2^ for UV-LED-embedded silicone tiles. These results indicate that the present operating point has substantial headroom for irradiance reduction. However, below a certain irradiance, cell replication and repair can outpace UV-induced damage, allowing biofilm establishment even at equivalent cumulative doses. Mohsin et al. observed this behavior below approximately 0.21 μW/cm^2^ under their experimental conditions [65]. Above this threshold, time-dose reciprocity holds and proportional irradiance-time tradeoffs should yield comparable performance during continuous exposure.

The present results support the use of UEG on small, optically sensitive surfaces, such as sensor windows and camera housing, where fouling directly compromises data quality. Although promising, operating points in Fig. S14 require direct experimental validation before adoption as design recommendations. The same principle of delivering UV from within the protected surface also holds promise for larger submerged structures, including ship hulls. Realizing this potential will require several considerations to be addressed before large-area deployment can be recommended. These include distributed power delivery across extended surfaces, cost-effective fabrication of UV-emitting substrate over large or curved areas, and long-term durability of the coatings, and LED assemblies under abrasion, impact, and flow-induced stress.

## CRediT authorship contribution statement

**Leila Alidokht:** Writing – review & editing, Writing – original draft, Visualization, Validation, Methodology, Investigation, Formal analysis, Data curation, Conceptualization. **Karina A. Chavarria:** Writing – review & editing, Writing – original draft, Validation, Methodology, Investigation, Data curation. **Dylan Eggers:** Writing – original draft, Visualization, Methodology, Investigation, Formal analysis, Data curation. **Kelli Hunsucker:** Writing – review & editing, Writing – original draft, Validation, Methodology, Investigation, Formal analysis. **Mariana Lanzarini-Lopes:** Writing – review & editing, Writing – original draft, Visualization, Validation, Supervision, Resources, Project administration, Methodology, Investigation, Funding acquisition, Formal analysis, Data curation, Conceptualization.

## Declaration of competing interest

The authors declare the following financial interests/personal relationships which may be considered as potential competing interests: Leila Alidokht reports financial support was provided by Office of Naval Research. Leila Alidokht has patent Ultraviolet Emitting Glass and Method of Making Thereof pending to UMA 23-0-53-01. If there are other authors, they declare that they have no known competing financial interests or personal relationships that could have appeared to influence the work reported in this paper.

## Data Availability

Data will be made available on request.

## Appendix A Supplementary data

Multimedia component 1 Additional information is available in Supporting Information, including UEG design and electrical specifications, thermal and mechanical performance evaluations of UEG housing, underwater optical simulations, field-site water quality characterization, operational duty-cycle selection, sample handling and shipping procedures, and supplementary microbial community analyses (alpha and beta diversity).
