# Supplementary material for: Multi-month fouling control on UV-emitting glass: performance, energy dependence, and insights into microbial community drivers
Source: Biofilm. 2026 Oct 27;12:100407. doi: 10.1016/j.bioflm.2026.100407 (PMC13743521; doi:10.1016/j.bioflm.2026.100407)
Supplement: Supplementary file 1 [file mmc1.docx]

**Supporting Information**

**Multi-month fouling prevention on UV-emitting glass: performance, energy dependence, and insights into microbial community drivers**

1. **UEG housing and electrical specifications of UEG**

The UEG housing consisted of two assembled components: a brass base containing six UV-LED mounts, with each LED starboard fastened to the brass base using screws and a 1 mm thick thermal paste layer at the LED-brass interface to promote heat dissipation, and a white PVDF cover section that housed the UEG lens and mated with the brass base. The two components were sealed using a greased O-ring and mechanically fastened to ensure a watertight assembly. Electrical wiring from the LED boards was consolidated and routed through the bottom of the brass base via a fully waterproof ruggedized connector with a threaded locking sleeve. The external cable connected to a dimmable LED driver (Violumas, San Diego, CA) powering six LEDs wired in serieswith each LED board rated at 6.4 V and 1 A, corresponding to a total supply of 45 V and 1 A under maximum operating conditions.

Control housing components were 3D-printed in nylon (PA2200) at the UMass ADDFab facility.

**2. Thermal simulation and hydrostatic pressure tests**

Thermal performance of the waterproof housing was evaluated by Design First Co. Canada, using conduction–convection simulations under conservative operating conditions. The model accounted for thermal resistance at all material interfaces, including low-conductivity thermal paste (k = 1 W/m/K) applied between the LED starboard and the housing, with paste thicknesses of 0.1 and 0.5 mm considered. Heat transfer through the LED assembly was represented by lumped thermal resistances for the brass base (1.25 K/W) and frame cover (0.06 K/W) interfaces, with a frame-cover contact area of 0.0089 m². Each LED was assumed to dissipate 5 W of heat under continuous operation at maximum power, with all electrical input converted to heat to represent a worst-case scenario. Simulations were conducted in a water environment at an ambient temperature of 32 °C, using a convection coefficient of 500 W/m^2^/K. The housing design was validated through a 24-hour hydrostatic pressure test performed by the manufacturer, confirming its watertight integrity at a simulated depth of 10 m. Although the specific test methodology was proprietary, the results verified that the enclosure remained leak-free under the prescribed conditions.

**3. Underwater ray-tracing simulations**

The simulations were performed by Violumas (San Diego, CA) to quantify the effects of the housing geometry and seawater on UV irradiance reaching the colonizable lens surface. The simulations used a Lambertian scattering model to represent the angular emission of the UV source, incorporated CAD files of the assembled housing and the experimentally measured irradiance data from the bench characterization and were run at source inputs of 80 mW (100%), 40 mW (50%), and 24 mW (30%) per LED. Refractive indices of 0.4619 and 1.4 were assigned to brass base and the PVDF cover frame, respectively, to account for their optical influence. Irradiance was evaluated at the coated lens surface (top) at detector distances of 0.02, 4.6, and 7.6 mm, and at the uncoated surface facing the housing interior (bottom) at 0.02 mm, under both ambient and submerged conditions. For the submerged case, the housing was placed within a cylindrical seawater volume on both sides of the geometry, with water optical properties defined by a refractive index of 1.35 at 265 nm and a UV transmission of 98.6% over a 10 mm path length.

4. **Water quality measurements in test site at Cape Marina, Port Canaveral, FL**

Turbidity data were collected weekly from May 2020 through August 2021 at 75 cm depth using a YSI ProDSS multiparameter sensor and reported in Formazin Nephelometric Units (FNU).

5. **Selection of the operational duty cycle**

Laboratory experiments were designed to compare continuous and intermittent UV operation while maintaining equivalent daily UV exposure, with the objectives of assessing biofilm control performance and minimizing thermal and operational stress on the LEDs. In these tests, UEG slides equipped with LED connections at one cross-sectional end were used. The slides were immersed in artificial seawater inoculated with marine bacteria and operated for 10 days at an average UV irradiance of 41 µW/cm². Details on Bacterial strain and chemical composition of the water matrix are described in our previous work (Alidokht et al., 2026). This irradiance level was chosen to closely approximate the UV output of the UEG lens while avoiding LED damage associated with excessive power input and heat accumulation. Three daily UV exposure durations, 5, 10, and 12 h/day, were evaluated. For each exposure duration, both continuous and intermittent irradiation schemes were examined to assess the influence of temporal UV distribution. For the 5 h/day exposure, UV irradiation was applied either as a single continuous 5 h period or as intermittent operation consisting of 150 cycles of 2 min On and 7.36 min Off. For the 10 h/day exposure, the tested schemes included a continuous 10 h on / 14 h off period and an intermittent schedule of 150 cycles of 4 min on and 5 min off. For the 12 h/day exposure, three schemes were evaluated: a continuous 12 h on / 12 h off period, 24 cycles of 30 min on and 30 min off, and 72 cycles of 10 min on and 10 min off. The outcomes of these laboratory experiments informed the selection of operating conditions for the submerged field deployment.

Results revealed that intermittent UV cycling significantly enhances biofilm prevention compared to continuous operation (Figure S6). Single cycle operations (5 hours on/19 hours off and 10 hours on/14 hours off) achieved modest LRV values of 1.09 and 1.39, respectively, indicating limited biofilm reduction effectiveness. In contrast, extended cycling significantly enhanced performance: 150-cycle regimes achieved LRVs of 1.79 (2 min on/7.36 min off) and 2.77 (4 min on/5 min off). The most effective performance was observed in the daily exposure protocols operating for 12 hours total, where 24 cycles (30 minutes on/30 minutes off) achieved an LRV of 2.38, and 72 cycles (10 minutes on/10 minutes off) reached the highest LRV of 2.87. Based on these results, the 30-minute on/30-minute off cycling protocol was selected for long-term performance testing because it provides acceptable biofilm prevention effectiveness while ensuring LED safety and longevity. We have previously observed that shorter intermittent cycles may cause LED degradation during extended operational periods.

Preliminary tests across continuous and intermittent UV exposure regimes showed that 12-hour daily protocols of repeated short cycles substantially outperformed long single cycles in biofilm prevention, consistent with UV-C being most effective against cells at or before attachment, when they remain unprotected by extracellular polymeric substances (Alidokht et al., 2026; Renner and Weibel, 2011). The 30 min on / 30 min off cycle was selected for long-term marine testing because it captured most of the prevention benefit while limiting the switching frequency, which in our prior observations accelerates LED degradation (Figure S6).

6. **Samples shipping to UMass Amherst laboratory**

Each retrieved lens was placed in a sterilized conical container with 2 mL of seawater, fouled surface oriented downward. The container opening matched the lens diameter to minimize movement during transport. Each container was sealed with a silicone cap and parafilm to prevent leakage and drying, placed in a cooler with ice packs, and shipped to UMass Amherst overnight.

**7. UV transmittance of the lens**

The optical transmittance of the coated lenses was evaluated using UV-Vis spectroscopy. The coated lenses exhibited visible light transparency (>380–759 nm) exceeding 80%, corresponding to a 13.4% reduction relative to the uncoated lenses. In comparison, our previous work(Alidokht et al., 2024) reported visible-region transmittance exceeding 95% for coated glass substrates. The lower transparency observed in the present study is attributed to the substantially greater coating thickness used in the current system. Specifically, the coating thickness in this study was approximately 7 µm, as specified by the manufacturer, whereas the coating thickness reported previously was 422 ± 83 nm. According to Rayleigh scattering theory (Novak, 1993), attenuation of light transmission in nanoparticle-polymer composite coatings arises from scattering phenomena, which increase with optical path length. Consequently, the increased coating thickness in the present study likely enhanced light scattering within the coating layer, leading to reduced optical transparency. The UV-Vis transmission spectra of the coated and uncoated lenses are presented in Figure S4.

**Figure S1**. Detailed dimensions of UEG housing.

**Figure S2**. 3D-printed holder accommodating the UEG lens, six UV LEDs mounted on individual heat sinks arranged around the lens perimeter, used for UV emission measurements.

a

b

**Figure S3**. CD Thermal Simulation Results with 0.1 mm (a) and 0.5 mm (b) thermal paste.

% Transmittance

| Wavelength (nm) | Plain lens | Coated lens |
| --- | --- | --- |
| 1100 | 86.0009 | 82.6367 |
| 1094.99 | 93.7832 | 95.4759 |
| 1089.99 | 92.9514 | 91.0443 |
| 1085 | 97.646 | 91.6772 |
| 1079.99 | 95.457 | 90.725 |
| 1074.99 | 96.5637 | 92.9236 |
| 1070.01 | 93.1334 | 90.923 |
| 1065.01 | 96.5069 | 92.3077 |
| 1059.99 | 95.4073 | 93.0588 |
| 1055.01 | 95.8005 | 93.0613 |
| 1049.99 | 95.7533 | 92.0996 |
| 1045.01 | 95.0239 | 93.6446 |
| 1040.01 | 96.0168 | 92.7656 |
| 1035 | 95.9994 | 92.8156 |
| 1030 | 96.1616 | 92.8815 |
| 1025.01 | 96.9168 | 93.6356 |
| 1020.01 | 97.0042 | 93.5256 |
| 1015 | 97.2634 | 93.9292 |
| 1010 | 97.2786 | 93.5001 |
| 1005.01 | 97.3361 | 93.736 |
| 1000.01 | 97.6834 | 93.025 |
| 994.995 | 97.4604 | 93.4226 |
| 989.996 | 97.1339 | 93.3592 |
| 985.013 | 97.5601 | 92.9618 |
| 979.985 | 97.4071 | 93.3084 |
| 975.005 | 97.2756 | 93.0934 |
| 970.011 | 97.3394 | 92.952 |
| 965.003 | 97.2526 | 92.9067 |
| 960.013 | 97.1375 | 92.8797 |
| 955.01 | 97.3788 | 92.5016 |
| 949.992 | 97.1495 | 92.4419 |
| 944.993 | 96.9003 | 92.5447 |
| 940.013 | 96.8813 | 92.4801 |
| 934.987 | 96.7254 | 92.2193 |
| 930.014 | 96.6683 | 91.8813 |
| 924.994 | 96.3862 | 91.6767 |
| 919.995 | 96.4641 | 91.4637 |
| 915.015 | 96.6539 | 91.614 |
| 909.99 | 96.437 | 91.15 |
| 904.986 | 96.1222 | 91.1631 |
| 900.002 | 96.2195 | 90.9555 |
| 895.006 | 96.3589 | 91.1144 |
| 889.998 | 95.8867 | 90.566 |
| 885.012 | 95.6725 | 90.3542 |
| 880.013 | 95.9202 | 90.562 |
| 875.003 | 95.6309 | 90.0644 |
| 870.015 | 95.2851 | 89.5654 |
| 865.015 | 95.2101 | 89.7925 |
| 860.004 | 94.7047 | 89.252 |
| 855.016 | 94.907 | 89.4218 |
| 850.016 | 94.5227 | 89.0804 |
| 845.006 | 94.566 | 88.9259 |
| 839.984 | 94.3036 | 88.8508 |
| 834.986 | 94.352 | 88.7223 |
| 830.011 | 94.3422 | 88.4905 |
| 824.991 | 94.3396 | 88.4587 |
| 819.995 | 94.2814 | 88.6406 |
| 814.989 | 94.432 | 88.335 |
| 810.007 | 94.1925 | 88.2987 |
| 805.016 | 94.1953 | 87.9343 |
| 800.013 | 94.2989 | 88.2619 |
| 795.001 | 94.3813 | 87.9985 |
| 790.014 | 94.6325 | 87.9656 |
| 784.983 | 94.5895 | 88.0568 |
| 780.011 | 94.4329 | 88.0584 |
| 774.995 | 94.6284 | 87.9263 |
| 770.005 | 94.6457 | 87.9082 |
| 765.005 | 94.9331 | 87.9789 |
| 759.996 | 94.9706 | 88.0918 |
| 755.013 | 95.1355 | 88.2175 |
| 749.985 | 95.6357 | 88.4197 |
| 744.984 | 95.8672 | 88.5268 |
| 740.01 | 96.1441 | 88.7654 |
| 734.991 | 96.5383 | 89.0543 |
| 729.999 | 96.967 | 89.5183 |
| 724.998 | 97.3834 | 89.5787 |
| 719.989 | 97.4743 | 89.5386 |
| 715.008 | 97.4099 | 89.441 |
| 710.017 | 97.0037 | 88.9771 |
| 704.983 | 96.3695 | 88.2344 |
| 700.013 | 95.4729 | 87.4054 |
| 694.998 | 94.8511 | 86.7237 |
| 690.011 | 94.2757 | 86.0648 |
| 685.017 | 93.8955 | 85.5539 |
| 680.014 | 93.4291 | 85.1008 |
| 675.004 | 92.9638 | 84.6006 |
| 669.986 | 92.7082 | 84.2395 |
| 664.997 | 92.8242 | 84.2669 |
| 660 | 92.8617 | 84.0896 |
| 654.996 | 93.041 | 83.9858 |
| 649.984 | 92.9214 | 83.9598 |
| 645.002 | 92.9628 | 83.7796 |
| 640.012 | 92.8611 | 83.6087 |
| 635.016 | 92.7708 | 83.5618 |
| 630.012 | 92.9703 | 83.4756 |
| 625.001 | 92.9085 | 83.2786 |
| 619.983 | 92.8866 | 83.1889 |
| 614.996 | 92.9128 | 83.0041 |
| 610.001 | 92.8989 | 82.8072 |
| 605 | 92.8386 | 82.7751 |
| 599.992 | 93.1131 | 82.6677 |
| 595.015 | 93.0818 | 82.5901 |
| 589.994 | 93.1082 | 82.523 |
| 585.004 | 93.2211 | 82.4711 |
| 580.008 | 93.326 | 82.3419 |
| 575.005 | 93.4967 | 82.328 |
| 569.996 | 93.7675 | 82.3585 |
| 565.019 | 94.0131 | 82.3786 |
| 559.997 | 94.1723 | 82.3858 |
| 555.008 | 94.4507 | 82.4326 |
| 550.012 | 94.5805 | 82.4253 |
| 545.011 | 94.7953 | 82.3539 |
| 540.003 | 95.0173 | 82.3204 |
| 534.99 | 94.7899 | 81.9393 |
| 530.009 | 94.4597 | 81.5022 |
| 524.985 | 94.3906 | 81.1809 |
| 519.993 | 93.8164 | 80.5311 |
| 514.995 | 93.4756 | 79.9447 |
| 509.992 | 93.1635 | 79.469 |
| 504.984 | 92.9091 | 79.0941 |
| 500.008 | 92.7306 | 78.6685 |
| 494.989 | 92.6205 | 78.3864 |
| 490.004 | 92.7399 | 78.1911 |
| 485.012 | 92.8455 | 77.9854 |
| 480.016 | 93.1167 | 77.9849 |
| 475.015 | 93.0678 | 77.7096 |
| 470.009 | 92.7836 | 77.15 |
| 464.999 | 92.2823 | 76.4355 |
| 459.983 | 91.9241 | 75.7857 |
| 455.001 | 91.4514 | 75.1469 |
| 450.014 | 91.2159 | 74.6723 |
| 444.985 | 91.1366 | 74.3015 |
| 439.989 | 91.0985 | 73.9287 |
| 434.988 | 91.1188 | 73.5969 |
| 429.984 | 91.2328 | 73.4117 |
| 425.013 | 91.4041 | 73.1656 |
| 420 | 91.658 | 73.0127 |
| 414.982 | 91.8514 | 72.7966 |
| 409.999 | 92.1882 | 72.6031 |
| 405.011 | 92.5686 | 72.4822 |
| 399.981 | 92.7122 | 72.2058 |
| 394.985 | 92.8476 | 71.8601 |
| 389.986 | 92.953 | 71.4602 |
| 384.983 | 92.9696 | 70.9825 |
| 380.014 | 92.9656 | 70.3857 |
| 375.003 | 92.9088 | 69.8824 |
| 369.988 | 92.5153 | 69.0996 |
| 365.009 | 92.4503 | 68.4981 |
| 359.986 | 92.3973 | 67.895 |
| 355 | 92.43 | 67.2178 |
| 350.009 | 92.5918 | 66.7667 |
| 345.016 | 92.8443 | 66.2977 |
| 340.018 | 93.0338 | 65.7686 |
| 335.018 | 93.3783 | 65.3638 |
| 330.014 | 93.72 | 64.8777 |
| 325.007 | 93.9876 | 64.2325 |
| 319.996 | 94.1309 | 63.4563 |
| 314.983 | 94.2802 | 62.7516 |
| 310.006 | 94.0986 | 61.8035 |
| 304.986 | 94.1412 | 61.0132 |
| 300.003 | 94.3893 | 60.1486 |
| 295.016 | 94.4722 | 59.3314 |
| 289.988 | 94.5826 | 58.3732 |
| 284.996 | 94.6941 | 57.3979 |
| 280.001 | 94.6743 | 56.4256 |
| 275.003 | 94.484 | 55.1762 |
| 270.003 | 94.3935 | 53.9581 |
| 265 | 93.9588 | 52.4279 |
| 259.995 | 92.6649 | 50.5274 |
| 254.987 | 88.3201 | 47.0424 |
| 250.016 | 82.1321 | 42.5466 |
| 245.003 | 76.8403 | 38.5831 |
| 239.988 | 75.267 | 36.4607 |
| 235.01 | 77.9604 | 36.2156 |
| 229.99 | 80.4004 | 35.7606 |
| 225.007 | 79.3051 | 33.7892 |
| 219.983 | 72.7828 | 29.248 |
| 214.996 | 59.0864 | 22.9382 |
| 210.007 | 45.8317 | 16.5688 |
| 205.017 | 36.3099 | 12.6526 |
| 199.984 | 31.8974 | 10.9368 |
| 194.989 | 29.9781 |  |
| 189.993 | 25.3882 |  |

**Figure S4**. UV-Vis transmission spectra of the coated and uncoated lenses.

**Figure S5**. UV irradiance heat map of UEG at different input power levels. The red circle denotes the region of the lens exposed to seawater during submersion testing. The values shown at the center of each heat map represent the average UV irradiance within the seawater-exposed region enclosed by the red circle.

**Figure S6**. Effect of cycling LED on enhancing biofilm prevention by UEG.

**Figure S7**. Visual progression of fouling on UEGs and controls and over submersion period. A USAF resolution target positioned behind the UEG glass within the housing provides an independent indicator of optical clarity.

Percent Cover by Organism

|  | Film | Hydriod | EB | AB | Barn | TW | Tunicate | Incipient |
| --- | --- | --- | --- | --- | --- | --- | --- | --- |
| Month 1 Control | 96 |  |  |  | 2 |  | 2 |  |
| Month 1 UEG | 10 |  |  |  |  |  |  |  |
| Month 2 Control | 88 |  |  |  | 1 | 5 | 4 | 2 |
| Month 2 UEG | 40 |  |  |  |  |  |  | 2 |
| Month 3 Control | 16 | 2 | 4 | 1 | 2 | 10 | 65 |  |
| Month 3 UEG | 15 |  |  |  |  |  |  |  |

**Figure S8**. Visual assessment of biofouling coverage over submersion time. EB: Encrusting Bryozoans, AB: Arborescent Bryozoans, Barn: Barnacles, TW: Tubeworms (Calcareous Polychaetes).

**Figure S9**. OCT surface topography images of UEG and control units during submersion period. Images for UEG month-2 are unavailable due to coating detachment during ex-situ imaging.

**Figure S10.** Brightfield microscopy images of UEG and control units during submersion period.

**Alpha Diversity Analysis**

Alpha diversity patterns differed between control and UEG biofilm communities. In the bacterial 16S rRNA dataset, control surfaces showed a progressive decline in Chao1 richness, Shannon diversity, and inverse Simpson diversity from Month 1 to Month 3. In contrast, UEG bacterial communities increased in richness and Shannon diversity over time, although diversity values remained lower than controls at corresponding timepoints (Figure S11a). This pattern suggests that untreated surfaces supported rapid early bacterial colonization followed by increasing dominance of fewer taxa, whereas UEG surfaces initially constrained bacterial colonization but became more diverse over time.

Eukaryotic 18S rRNA alpha diversity showed a different treatment pattern. Control surfaces had higher Chao1 richness than UEG surfaces at comparable timepoints, but Shannon and inverse Simpson diversity declined sharply after Month 1, indicating increasing dominance by a small number of eukaryotic taxa. UEG eukaryotic communities increased in richness and diversity from Month 2 to Month 3 and maintained higher Shannon and inverse Simpson diversity than controls at these later timepoints (Figure S11b). These trends are consistent with reduced dominance by individual eukaryotic taxa on UEG surfaces. Because each condition and timepoint was represented by a single sample, these alpha diversity patterns are interpreted descriptively rather than statistically.

**Figure S11.** Alpha diversity of bacterial and eukaryotic biofilm communities. Chao1 richness, Shannon diversity, and inverse Simpson diversity are shown for bacterial communities based on 16S rRNA metabarcoding and eukaryotic communities based on 18S rRNA metabarcoding across the three-month deployment. The Month 1 UEG eukaryotic sample failed to sequence due to low biomass and is not shown.

Venn analysis was used to compare genus-level overlap between control and UEG communities. For bacterial communities, 68 genera were shared between control and UEG samples across the deployment, whereas 129 genera were unique to controls and 61 were unique to UEG samples (Figure S12a). The larger control-unique pool indicates broader bacterial colonization on untreated surfaces, while the smaller UEG-unique pool suggests that UEG operation restricted colonization to a more specific subset of bacterial taxa, consistent with previous observations on treated surfaces (Chambers et al., 2006; Naik et al., 2022; Qian et al., 2007).

For eukaryotic communities, genus-level overlap differed between treatments and changed over time. At Month 2, 29 genera were shared between control and UEG samples, with 44 genera unique to controls and 21 unique to UEG. By Month 3, 38 genera were shared, with 83 unique to controls and 40 unique to UEG samples (Figure S12b). The increase in unique genera over time on both treatments is consistent with continued community development (Richard et al., 2025; Whitworth et al., 2023). However, the consistently larger control-unique pool indicates broader eukaryotic colonization on untreated surfaces, whereas UEG surfaces supported a more restricted eukaryotic assemblage.

**Figure S12**. Venn diagrams showing shared and unique taxa in (A) bacterial and (B) eukaryotic biofilm communities across Months 1-3 of the experiment.

**Beta Diversity Analysis**

**UEG samples cluster more tightly than controls in beta diversity ordination.** Non-metric multidimensional scaling (NMDS) ordination of Bray-Curtis dissimilarity revealed partial separation between control and UEG samples in both the bacterial and eukaryotic communities (Figure S12). In the bacterial ordination (Figure S13a), UEG samples were positioned closer to one another in NMDS space than control samples, which showed greater dispersion. A similar pattern was observed for eukaryotic communities (Figure S13b), where UEG samples clustered more closely than controls despite some overlap between treatments. The relative proximity of UEG samples across both domains is consistent with a tendency toward more homogeneous community composition under UEG conditions, paralleling the reduced taxonomic dominance and greater evenness described above. Overall, these results indicate that *Pseudoalteromonas* increasingly dominates biofilm communities over time in control lenses, while UEG treatment appears to suppress its dominance, allowing a more even distribution of other bacterial taxa.

**Figure S13.** Non-metric multidimensional scaling (NMDS) ordination of beta diversity for (A) bacterial and (B) eukaryotic biofilm communities. Points represent individual samples and are colored by sample type. Ellipses indicate visual enclosures of sample groups in ordination space. Ordination patterns are interpreted descriptively due to the limited number of samples.

**Figure S14**. Projected daily energy budget of the UEG system as a function of operational irradiance at varying input power levels.

**References**

(1)	Alidokht, L.; Chavarria, K. A.; Lanzarini-Lopes, M. Optimizing Energy Budget for UV Driven Biofilm Prevention Using Response Surface Methodology. *Environ. Technol. Innov.* **2026**, *41*, 104742. https://doi.org/https://doi.org/10.1016/j.eti.2025.104742.

(2)	Renner, L. D.; Weibel, D. B. Physicochemical Regulation of Biofilm Formation. *MRS Bull.* **2011**, *36* (5), 347–355. https://doi.org/10.1557/mrs.2011.65.

(3)	Naik, A.; Smithers, M.; Moisander, P. H. Impacts of UV-C Irradiation on Marine Biofilm Community Succession. *Appl. Environ. Microbiol.* **2022**, *88* (4), e02298-21. https://doi.org/10.1128/aem.02298-21.

(4)	Chambers, L. D.; Stokes, K. R.; Walsh, F. C.; Wood, R. J. K. Modern Approaches to Marine Antifouling Coatings. *Surf. Coat. Technol.* **2006**, *201* (6), 3642–3652. https://doi.org/https://doi.org/10.1016/j.surfcoat.2006.08.129.

(5)	Qian, P.-Y.; Lau, S. C. K.; Dahms, H.-U.; Dobretsov, S.; Harder, T. Marine Biofilms as Mediators of Colonization by Marine Macroorganisms: Implications for Antifouling and Aquaculture. *Marine Biotechnology* **2007**, *9* (4), 399–410. https://doi.org/10.1007/s10126-007-9001-9.

(6)	Richard, K. N.; Hunsucker, K. Z.; Swain, G.; Kardish, M. R. Comparing the Effectiveness of UV-C on Dynamically Formed Field Biofilms. *Microorganisms* **2025**, *13* (11), 2561. https://doi.org/10.3390/microorganisms13112561.

(7)	Whitworth, P.; Clare, A. S.; Finlay, J. A.; Piola, R. F.; Plummer, J.; Aldred, N. Long-Term Ultraviolet Treatment for Macrofouling Control in Northern and Southern Hemispheres. *J. Mar. Sci. Eng.* **2023**, *11* (12). https://doi.org/10.3390/jmse11122211.
